# Cochrane highlights

**DOI:** 10.1590/S1516-31802009000400001

**Published:** 2009-12-07

**Authors:** Rachel Riera, Álvaro Nagib Atallah

**Affiliations:** 1 MD, MSc. Research assistant at the Brazilian Cochrane Center and editorial advisor of the São Paulo Medical Journal and of the journal Diagnóstico & Tratamento of the Associação Paulista de Medicina.; 2 MD, MSc, PhD. Director of the Brazilian Cochrane Center. Editor-in-chief of the São Paulo Medical Journal and of the journal Diagnóstico & Tratamento of the Associação Paulista de Medicina. Titular professor of Evidence-Based Medicine and Emergency Medicine of Universidade Federal de São Paulo - Escola Paulista de Medicina (Unifesp-EPM). E-mail: atallahmbe@uol.com.br

Systematic reviews and meta-analyses of good-quality studies are considered to be the best level of evidence for healthcare decision-making.^1^ To minimize bias and give consistency to the results, the methodology for these reviews is detailed and clear, and studies are only included after judicious critical evaluation. These results may assist clinicians, decision-makers and researchers who are evaluating new healthcare technologies. What is missing is widespread dissemination of this important source of information, with the aim of providing quality knowledge for the Brazilian population.

Starting from this issue, the São Paulo Medical Journal/Evidence for Health Care and the journal Diagnóstico & Tratamento will have a section for disseminating short abstracts of systematic Cochrane reviews of relevance to their readers. This initiative follows the example of several other prestigious international medical journals, such as Stroke: Journal of the American Heart Association, Journal of Emergency Primary Health Care, Bandolier Journal, Schizophrenia Bulletin and Anesthesia and Analgesia. Stroke, for example, in its issue of March 2009, carried a summary of the review “Interventions for deliberately altering blood pressure in acute stroke”.^2^

## Interventions to prevent the spread of the influenza virus

Inevitably, our inaugural section covers two reviews on influenza prevention: “Interventions for the interruption or reduction of the spread of respiratory viruses” and “Vaccines for preventing influenza in the elderly”. There are many other systematic Cochrane reviews with relevant evidence on different aspects of influenza prevention and treatment, and these are available free of charge from the international website of the Collaboration (http://www.cochrane.org) or from the website of the Brazilian Cochrane Center (http://www.centrocochranedobrasil.org.br/).

The selection of abstracts will be made from a list prepared by the Editorial Group of the Cochrane Collaboration, containing high-impact reviews that have been published in one of the latest electronic issues from the Cochrane Library. To help us in this selection, we invite you, the reader, to collaborate in this by suggesting topics that would be of interest to you, for our forthcoming issues.

It is well known that, to keep up-to-date, clinicians would need to read dozens of papers every day. It would be an impossible mission. And even if they had the time, they would also have to have skills for searching for and critically assessing the literature that are superior to those available through the teaching of normal curricular programs. On the other hand, a systematic review that takes years of work by two or three authors, with critical editorial supervision by three or four international editors, starts by identifying hundreds of papers of relevance for the matter in question, which are then structured, turned into synthesis form and summarized in terms of the subjects best studied, thereby resulting in not more than a dozen articles that can be summed in the form of a meta-analysis if appropriate.

Thus, reading a systematic review signifies incorporating the distilled knowledge from hundreds of articles on the subject. In other words, this is truly a form of magic within continuing education that enables readers to constantly keep up to date, in real time.

This new section will certainly provide a fundamental step forward for physicians, patients, managers, teachers and researchers who wish to receive quality knowledge and escape from the growing noise of excesses of poor-quality information, particularly from the internet.

Enjoy reading it.
